# Interstitial Lung Disease Is a Major Characteristic of Patients Who Test Positive for Anti-PM/Scl Antibody

**DOI:** 10.3389/fmed.2021.778211

**Published:** 2022-01-18

**Authors:** Yongpeng Ge, Xiaoming Shu, Linrong He, Chunjia Li, Xin Lu, Guochun Wang

**Affiliations:** Department of Rheumatology, China-Japan Friendship Hospital, Beijing, China

**Keywords:** anti-PM/Scl antibodies, interstitial lung disease, dermatomyositis, polymyositis, systemic sclerosis

## Abstract

**Objective:**

This study aimed to analyze the clinical features of anti-PM/Scl antibodies in Chinese patients.

**Method:**

We reviewed the clinical data of anti-PM/Scl antibody-positive patients, including their long-term follow-up.

**Results:**

A total of 30 patients carried anti-PM/Scl antibodies, 21 (70%) were females, and the mean age was 55.4 years, 15 (50%) and 10 (33.3%) patients were positive for anti-PM/Scl-75 and anti-PM/Scl-100, respectively. Fifteen cases (50%) were diagnosed as inflammatory myopathy, namely, 11 dermatomyositis (DM) and 4 polymyositis (PM). Five (16.7%) patients were diagnosed with overlap syndrome, and only one (3.3%) was diagnosed as systemic sclerosis. The other 9 (30%) patients were classified as undifferentiated connective tissue disease. Twenty-six (86.7%) had interstitial lung disease (ILD) and was the sole manifestation in 8 (26.7%) patients, 15 (58.0%) showed non-specific interstitial pneumonia based on high-resolution CT or lung biopsy. The majority of patients (95%) with mild and moderate groups on basis of pulmonary function tests. Compared to the anti-PM/Scl-100 group, the occurrence of clinical characteristics was not significantly different from the anti-PM/Scl-75 group, except the levels of C-reactive protein and erythrocyte sedimentation rate in the anti-PM/Scl-75 antibody-positive group were higher (*p* < 0.05). All patients with positive Ro-52 antibodies had ILD and were more likely to develop skin rash in the group with Ro-52 (*p* = 0.024). With a follow-up of the present cohort, 70.8% improved with treatment, but 16.7% of patients are easy to relapse.

**Conclusion:**

The anti-PM/Scl antibody occurred frequently in DM/PM patients, ILD was the major clinical feature, especially in patients combined with Ro-52. Some patients may complicate with ILD alone without extrapulmonary manifestations. Anti-PM/Scl antibodies positive patients were responsive to treatment.

## Introduction

A characteristic feature of patients with connective tissue diseases (CTDs) is the presence of autoantibodies in their sera that target intracellular components. Autoantibodies often characterize patients with distinct clinical features and often have different prognoses. Anti-PM/Scl antibodies were first described in patients with overlap syndrome of polymyositis (PM) and scleroderma (Scl) or systemic sclerosis (SSc) (1, 2). Anti-PM/Scl antibodies are a heterogeneous group of autoantibodies directed to several proteins of the nucleolar PM/Scl macromolecular complex. The two main autoantigenic protein components were identified and termed PM/Scl-75 and PM/Scl-100 based on their apparent molecular weights (3, 4).

Anti-PM/Scl antibodies have great diversity in their prevalence and clinical characteristic among different races and countries. A study showed that the anti-PM/Scl was found in 31% of PM/SSc patients, 11% of dermatomyositis (DM) patients, 8% of patients with PM alone, and 2% of SSc patients (5). De Lorenzo et al. reported muscle weakness, mechanic's hands, Raynaud syndrome, and interstitial lung disease (ILD) occurred frequently in anti-PM/Scl-positive patients (6). However, few reports have described cases with anti-PM/Scl antibodies in China. The clinical features, and differences between anti-PM/Scl-75 and PM/Scl-100, were previously unknown. To address these questions, we initially identified Chinese patients with the anti-PM/Scl antibody, reviewed all of their clinical data including response to treatment with follow-up, then compared the differences between patients with anti-PM/Scl-75 and PM/Scl-100.

## Methods and Materials

### Patients

Anti-PM/Scl antibodies were screened from the immune laboratory, with a total of about 5,000 samples between May 2015 and Oct 2020, and about 300 serum samples were from the ILD service. The following data of patients with anti-PM/Scl antibodies were obtained from the medical records: age, gender, pulmonary function tests (PFTs), electrocardiogram, ultrasonic cardiogram, pulmonary high-resolution CT (HRCT), levels of creatine kinase (CK) and lactate dehydrogenase (LDH), C-reactive protein (CRP), erythrocyte sedimentation rate (ESR), ferritin, immunoglobulin, presence of myositis-associated antibodies, and other myositis-specific autoantibodies (MSAs). If anti-PM/Scl-positive patients were combined with other MSAs, they were excluded. Response to treatment was documented, including complete follow-up on all patients.

Pulmonary function test (PFT) abnormalities manifested by restrictive changes [forced vital capacity (FVC) < 80% and diffusing capacity of carbon monoxide (DLCO) < 70% predicted]. The diagnosis of ILD depended on HRCT and/or lung biopsy. The ILD patterns were classified as usual interstitial pneumonia (UIP), organizing pneumonia (OP), non-specific interstitial pneumonia (NSIP), and lymphocytic interstitial pneumonia (LIP).

Patients with ILD were divided into three groups according to the initial PFT values in reference to the previous studies: the mild group (% predicted FVC > 75% and % predicted DLCO > 55%), the moderate group (% predicted FVC from 75 to 50% or %predicted DLCO from 55% to > 35%), and the severe group (% predicted FVC < 50% and % predicted DLCO < 35%) (7, 8).

According to the consensus statement of the American Thoracic Society on idiopathic pulmonary fibrosis, a decrease or increase of ≥10% in FVC was considered deterioration or improvement, respectively; either a decrease or increase of <10% in FVC was considered stable (9).

### Statistical Analysis

Analyses were performed with IBM SPSS software (Statistics for Windows, version 21.0) and *p* < 0.05 was considered to be statistically significant. Quantitative variables are reported as means and were compared using a non-parametric test. Categorical variables were reported as numbers and/or percentages and were compared using the chi-squared test or, when appropriate, the Fisher's exact test.

## Results

### Characteristics of Anti-PM/Scl Antibody-Positive Chinese Patients

**Figure 1:**
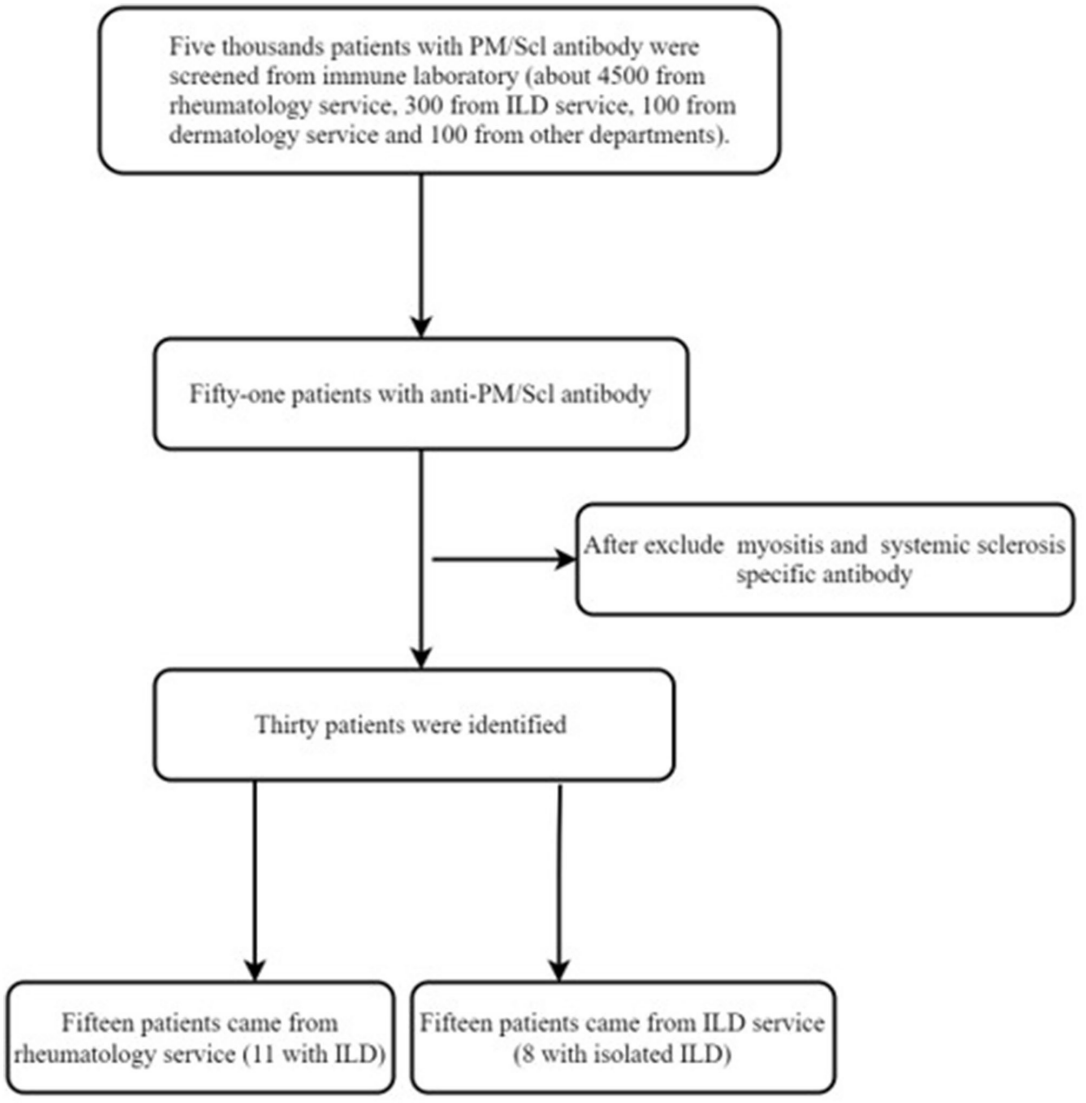
Selection flowchart.

A total of 30 patients were discovered by immunoblotting to be carrying anti-PM/Scl antibodies (see Figure 1). There were 21 (70%) females and 9 (30%) males among the patients carrying the anti-PM/Scl antibody. All patients were over 30 years old and the mean age was 55.4 years (range 32–83 years). Among these patients with anti-PM/Scl antibodies, 15 (50%) patients were positive only for anti-PM/Scl-75, 10 (33.3%) patients were positive only for anti-PM/Scl-100, and 5 (16.7%) patients were positive for both anti-PM/Scl-75 and anti-PM/Scl-100.

**Figure 2:**
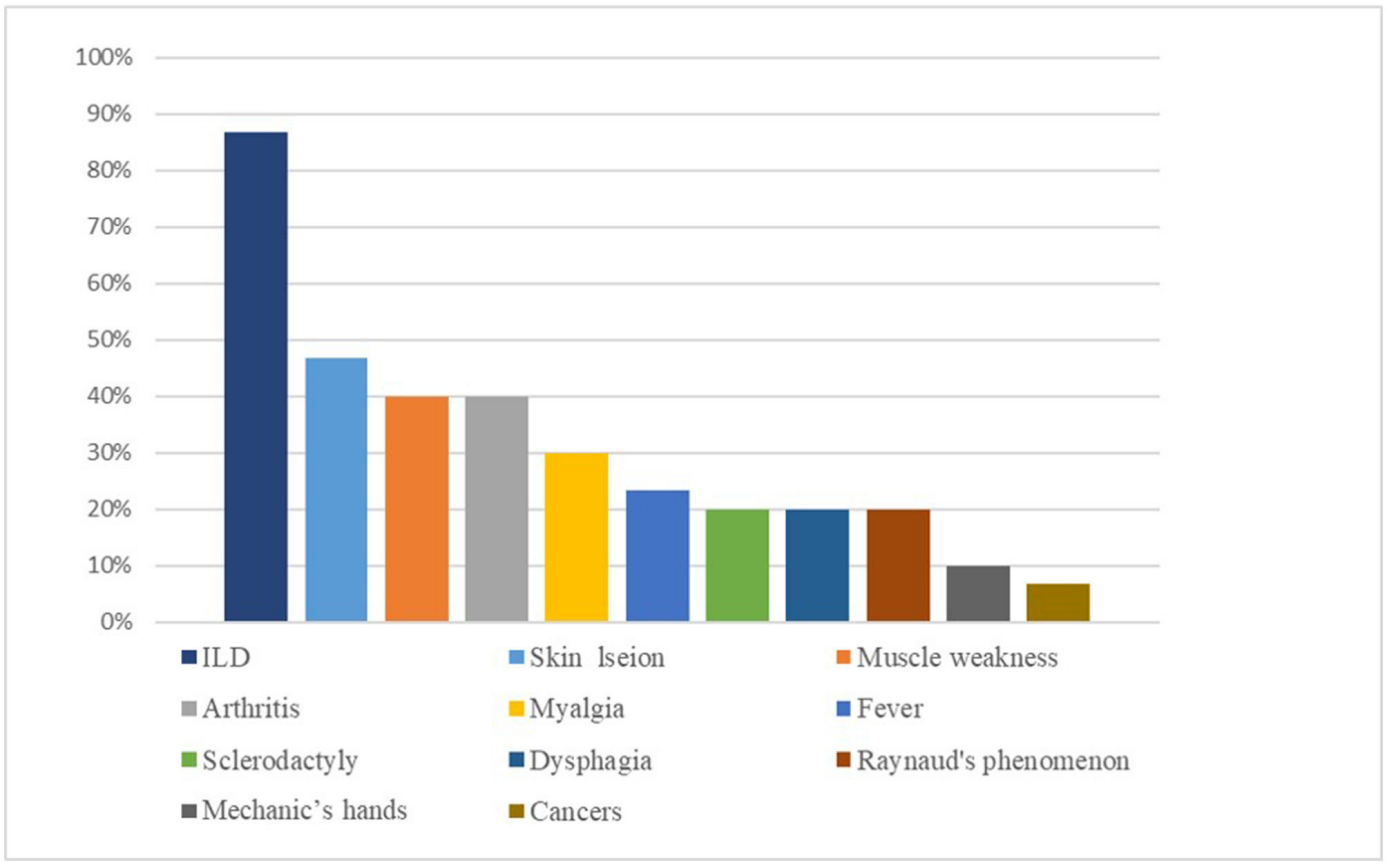
The incidence of various clinical features in patients who were positive for anti-PM/Scl antibody.

Muscle weakness and arthritis presented in 12 (40%) patients, and 9 (30%) suffered myalgia. In addition, 14 (46.7%) presented with the hallmark cutaneous manifestations of DM (Gottron's sign and heliotrope sign), sclerodactyly in 6 patients (20%), 3 (10%) demonstrated classic features of mechanic's hands, fever was present in 7 (23.3%), 6 (20%) suffered dysphagia, and Raynaud's phenomenon occurred in 6 (20%) patients. In addition, cancers were observed in two (6.7%) patients. Twenty-four (80%) patients were antinuclear antibodies-positive, and the titers ranged from 1:40 to 1:320 (nuclear or cytoplasmic speckled). Twelve (40%) patients tested positive for Ro-52 antibodies. The frequency of clinical features is listed in Figure 2.

A total of fifteen cases (50%) were diagnosed as myositis, namely, 11 DM and 4 PM. Five patients (16.7%) were diagnosed with overlap syndrome of myositis (3 DM and 2 PM) and SSc, and only one (3.3%) was diagnosed as SSc. The other 9 (30%) patients were classified as undifferentiated connective tissue disease (UCTD).

### Lung Involvement in Patients With Anti-PM/Scl Antibodies

**Figure 3:**
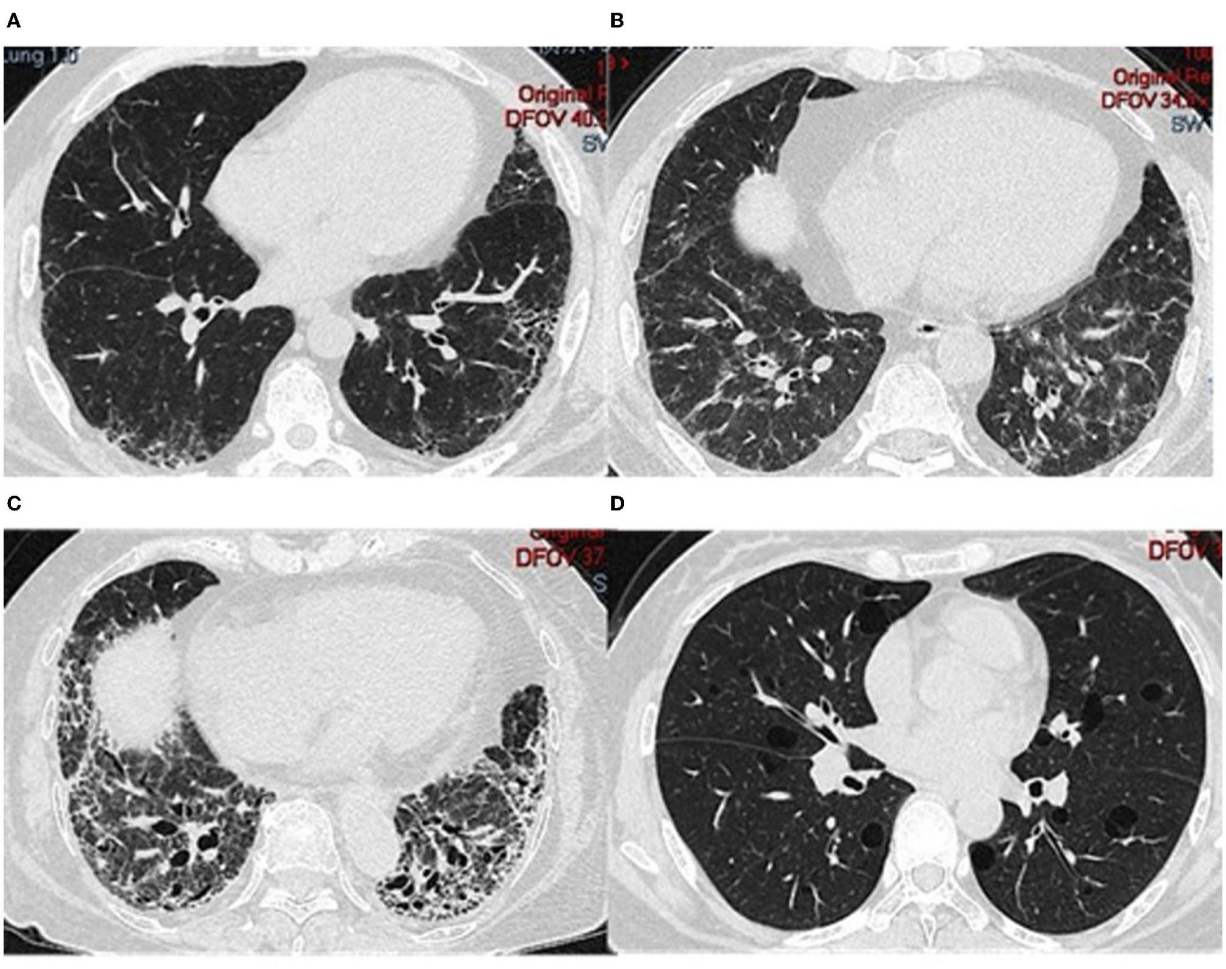
HRCT from different patients who tested positive for anti-PM/Scl. **(A)** 41-year-old man; **(B)** 52-year-old man; **(C)** 83-year-old woman; **(D)** 41-year-old woman.

A total of 26 (86.7%) patients were diagnosed with ILD by lung HRCT; cough and/or dyspnea were the first symptoms in 15 (50%) patients. In addition, ILD was the sole manifestation in 8 (26.7%) patients. Three (10%) patients experienced mild pulmonary arterial hypertension. The pattern of ILD was evaluated by HRCT or lung biopsy: 15 (58.0%) NSIP, 5 (19.2%) patients with UIP, 4 (15.4%) with OP, NSIP with OP overlap in one patient (3.8%), and 1 (3.8%) with LIP. Representative HRCT images are shown in Figure 3.

The PFTs were available from 20 patients, 10 (50%) patients were included in the mild group, 9 (45%) patients were included in the moderate group, and 1 (5%) patient in the severe group according to the basis of the PFT results.

### Clinical Difference Between Anti-PM/Scl-75 and Anti-PM/Scl-100

The average age of onset among anti-PM/Scl-75 antibody-positive patients was not different from patients with anti-PM/Scl-100 antibodies (*p* > 0.05). The occurrence of skin rashes, Raynaud's phenomenon, arthritis, fever, mechanic's hands, and cancer in the anti-PM/Scl-75 antibody-positive group were not significantly different from the anti-PM/Scl-100 group (*p* > 0.05). Levels of serum muscle enzymes—namely, CK and LDH in anti-PM/Scl-75 antibody-positive patients were not higher than those in anti-PM/Scl-100 patients (*p* > 0.05). As for the involvement of internal organs, the frequency of ILD was similar in both groups (*p* > 0.05). Although the frequency of muscular weakness and dysphagia in anti-PM/Scl-75 patients was higher than those in the anti-PM/Scl-100 group, the differences were not statistically significant (*p* > 0.05). CRP levels and ESR in anti-PM/Scl-75 patients were significantly higher than those in the anti-PM/Scl-100 group (*p* < 0.05) (see Table 1).

**Table 1:** Differences between patients who tested positive for anti-PM/Scl-75 and anti-PM/Scl-100.

**Characteristic**	**Patients with anti-PM/Scl-75**	**Patients with anti-PM/Scl-100**
N	15	10
Female/Male	11/4	6/4
Age (years)	56.9 ± 10.7	56.1 ± 11.6
Skin rash	6/15 (40%)	5/10 (50%)
Mechanic's hands	2/15 (13.3%)	1/10 (10%)
Muscular weakness	7/15 (46.7%)	2/10 (20%)
Dysphagia	3/15 (20%)	0/10 (0%)
Fever	4/15 (26.7%)	1/10 (10%)
Arthritis	5/15 (33.3%)	4/10 (40%)
Raynaud's phenomenon	1/15 (6.7%)	3/10 (30%)
ILD	13/15 (86.7%)	8/10 (80%)
Cancer	2/15 (13.3%)	0/10 (0%)
Ro-52	5/15 (33.3%)	3/10 (30%)
AST (normal < 40 U/L)	45.1 ± 48.0	20.2 ± 9.4
ALT (normal < 40 U/L)	37.4 ± 53.9	21.7 ± 10.6
CK (normal < 200 U/L)	350.0 ± 906.6	117.7 ± 148.0
LDH (normal < 250 U/L)	474.9 ± 421.1	231.6 ± 69.0
CRP (normal < 0.8 mg/dL)	1.7 ± 2.1	0.4 ± 0.3^*^
ESR (normal < 20 mm/h)	33.4 ± 31.3	12.1 ± 11.4^*^

*LDH, lactate dehydrogenase; ALT, alanine transaminase; AST, aspartate transaminase; ILD, interstitial lung disease;*

* *p < 0.05*.

### Clinical Difference Between Anti-PM/Scl Patients With and Without Ro-52

Among these patients with anti-PM/Scl antibodies, the occurrence of skin rashes in the Ro-52 antibody-positive group were significantly higher than the group without Ro-52 (*p* = 0.024), but Raynaud's phenomenon, arthritis, fever, mechanic's hands, and muscular weakness in the Ro-52 group were not significantly different from the group without Ro-52 (*p* > 0.05). The frequency of ILD in Ro-52 patients was similarly higher than those in the group without Ro-52, but the differences were not statistically significant (*p* > 0.05) (see Table 2).

**Table 2:** Differences between the anti-PM/Scl patients with and without Ro-52.

**Characteristic**	**Ro-52-positive**	**Ro-52-negative**
*N*	12	18
Female/Male	9/3	12/6
Skin rash	9/12 (75%)^*^	5/18 (27.8%)
Mechanic's hands	2/12 (16.7%)	1/18 (5.6%)
Muscular weakness	7/12 (58.3%)	5/18 (27.8%)
Fever	4/12 (33.3%)	3/18 (16.7%)
Arthritis	5/12 (41.7%)	7/18 (38.9%)
Raynaud's phenomenon	2/12 (16.7%)	3/18 (16.7%)
ILD	12/12 (100%)	14/18 (77.8%)

* *p < 0.05*.

### Follow-Up Study of Anti-PM/Scl Antibody-Positive Patients

**Figure 4:**
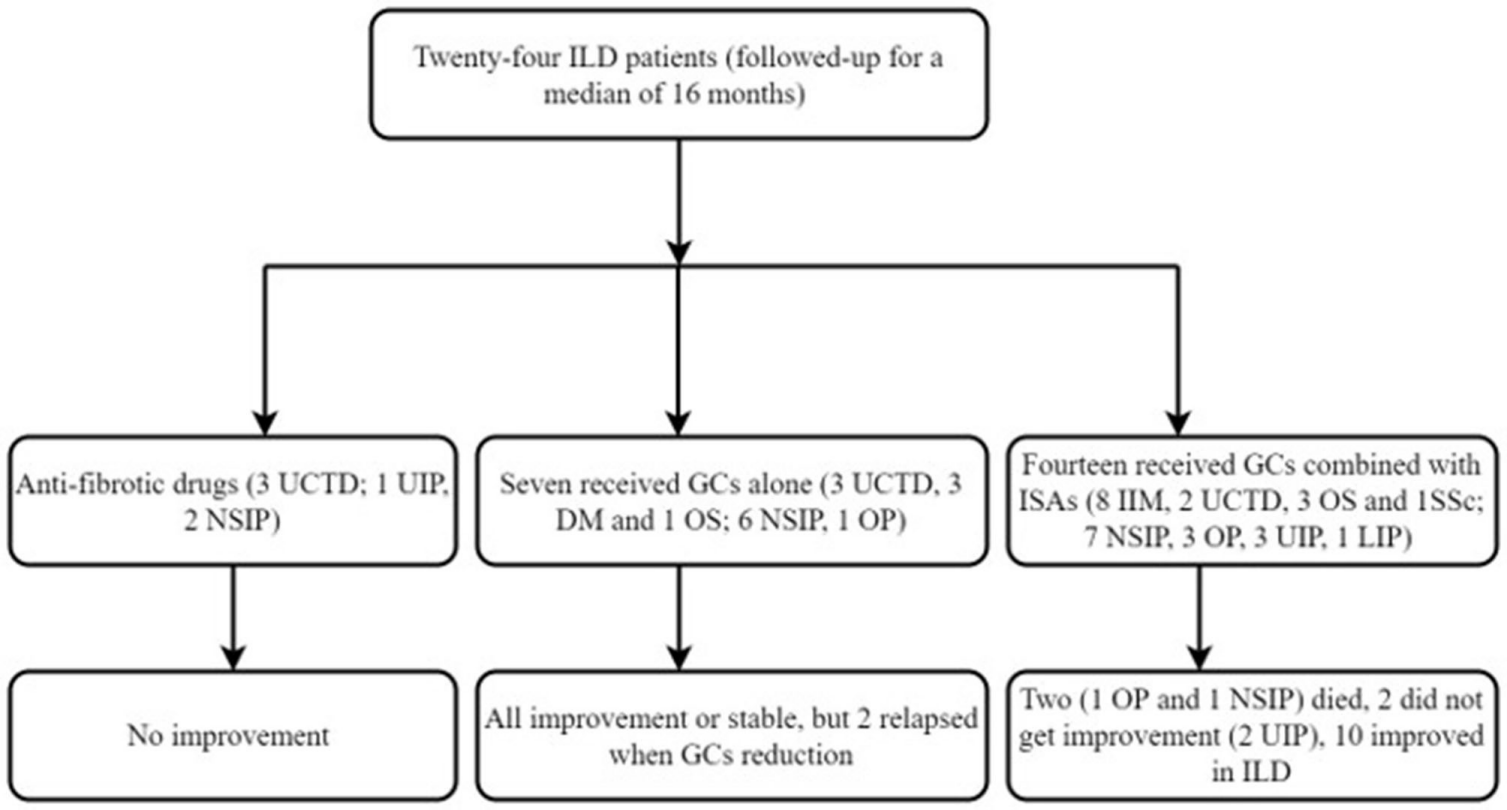
Fellow up of patients with ILD. GCs, glucocorticoids; OS, overlapping syndrome; ISA, immunosuppressive agents.

**Figure 5:**
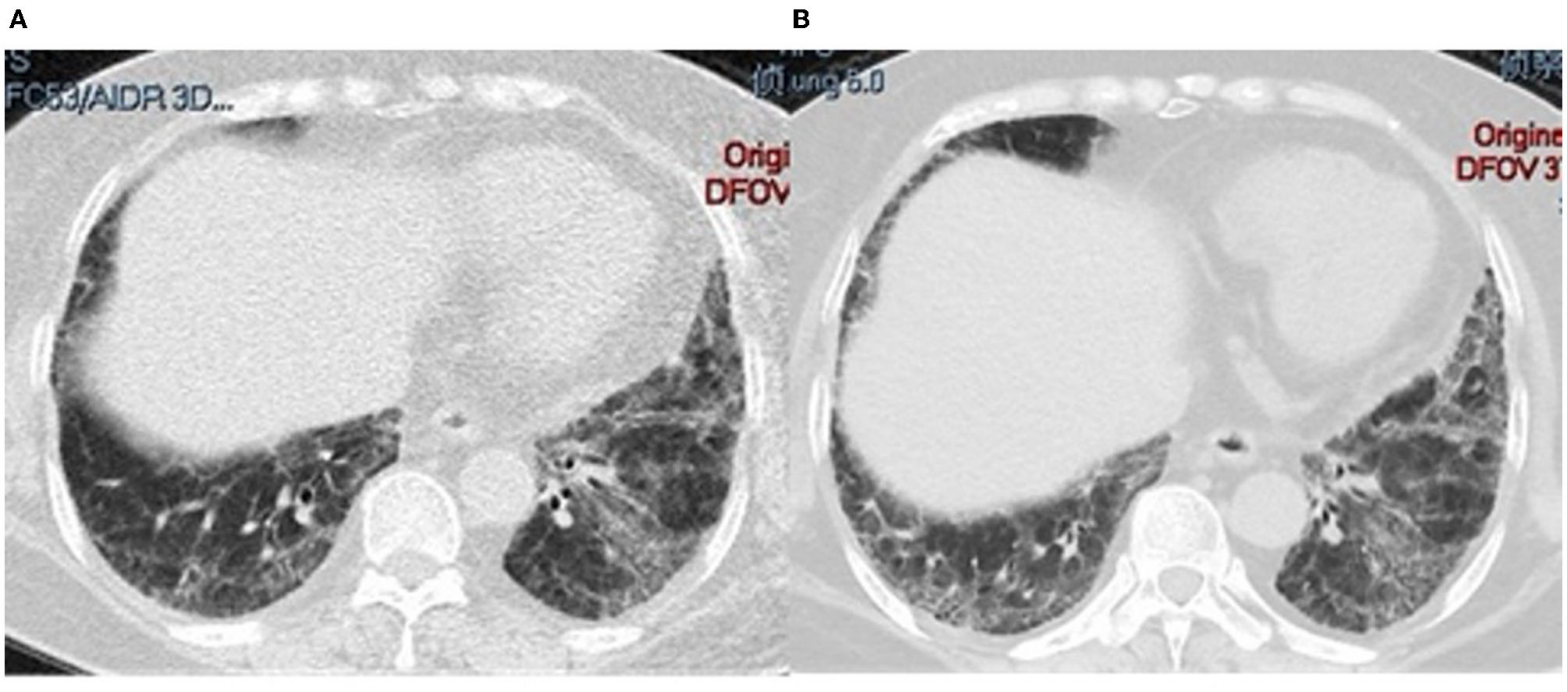
Lung CT changes in a 41-year-old man with PM/Scl-75 and−100. **(A)** CT at onset; **(B)** ILD progression after 8 months of treatment with an anti-fibrotic drug.

Five patients with ILD did not receive any immunosuppressive treatments except for the antifibrotic drug (pirfenidone). Eight patients received glucocorticoids alone, and the remaining 17 patients received a combination of immune agents such as cyclophosphamide, Tripterygium Wilfordii Polyglycoside, and cyclosporine A. Twenty-four patients with ILD were followed for a median of 16 months (range, 4–51 months) (Figure 4). Among these patients, two (8.3%) patients died in the follow-up; the reasons for death were ILD aggravated and severe pulmonary infection. Thirteen (54.1%) patients with ILD got improvement or stable after treatment; although two patients achieved improvement, there were recurrent infections, such as bacterial pneumonia Epstein-Barr virus and cytomegalovirus infection. Four (16.7%) patients got improvement in muscle weakness and ILD, but often relapsed when glucocorticoid was reduced to about 15 mg daily. Two (8.3%) patients with immunotherapy had no improvement; both of them had ongoing ILD despite antilung fibrosis therapy (Figure 5). In addition, 3 (12.5%) patients with ILD did not get improvement after antifibrotic drugs.

## Discussion

This is the first retrospective study to describe the clinical feature of anti-PM/Scl antibodies in Chinese patients. All the patients developed the disease after the age of 30 years, mainly seen in female patients, and lung involvement was the principal prominent complication. Half of the patients presented with DM. Muscle involvement and arthritis were seen in some patients, and the main pulmonary pathological changes of ILD were NSIP. Fortunately, most patients responded well to immunosuppressive treatment and achieved a good outcome.

Polymyositis (PM)/Scl-75 and PM/Scl-100 are the two main immune targets in anti-PM/Scl antibodies. The frequency of anti-PM/Scl also appears to vary between different ethnic groups. Most patients with anti-PM/Scl antibodies were double positive for anti-PM/Scl-75 and anti-PM/Scl-100 (6, 10). De Lorenzo et al. reported that among patients with anti-PM/Scl autoantibodies, 63% were positive for both anti-PM/Scl-75 and anti-PM/Scl-100, 20% were positive only for anti-PM/Scl-100, and 17% were positive only for anti-PM/Scl-75 (6). Recently, 11 Japanese patients with anti-PM/Scl antibody were summarized by Nakamura et al., 6 patients were positive for both anti-PM/Scl-75 and anti-PM/Scl-100, 4 only for anti-PM/Scl-100, and one positive for anti-PM/Scl-75 alone (10). Contrast to previous studies, this study showed half of the patients with anti-PM/Scl-75 along, a third of patients were positive only for anti-PM/Scl-100, and very few patients with positive double antibody. In addition, previous studies had suggested that anti-PM/Scl antibodies were mainly present in patients with overlap syndrome, and rarely in patients with DM/PM alone (5). In the series of Raijmakers et al., only three of 88 patients with isolated PM/DM had anti-PM-Scl antibodies (11). But a study from Japan showed that 9 patients were positive for anti-PM/Scl antibodies, namely, 4 patients with UCTD, 3 patients with DM, 1 with SSc, and 1 with Sjögren's syndrome (12). Unlike previous reports, half of the patients with anti-PM/Scl antibodies showed symptoms of DM/PM, and only a few of the patients belong to overlap syndrome and SSc in this study. These differences may be due to different races and ethnic backgrounds.

In addition, the frequency of other clinical features varies from different studies. Raynaud's phenomenon (50–78%), joint involvement (78–86%), muscle weakness (51–93%), dysphagia (21–56%), and mechanic hands (75–80%) were very common in patients with anti-PM/Scl antibodies (6, 13–16). But in our cohort, these characteristics were relatively infrequent except the muscle and joint involvement.

In this study, 2 anti-PM/Scl antibody-positive patients we diagnosed with malignant tumors. Other studies also have similar findings. Muro et al. reported that internal malignancy (pharynx and prostate) was also recognized in two patients 3 years before or after disease onset (12). Among the 20 patients with anti-PM/Scl antibodies, three with cancer. Cancer onset preceded 2 years of initial clinical signs of DM in one patient, while cancer developed after PM/DM diagnosis in the two remaining patients (6 months; 7 years) (17). Bruni et al. described a 43-year-old woman with pancreatic tumor and clinical features of SSc and myositis associated with PM/Scl antibodies. After resection of the tumor, clinical symptoms of the patient were relieved (18). Therefore, patients with anti-PM/Scl antibodies should also be alert for the development of tumors. However, it is unclear whether the malignancies in these patients are related to anti-PM/Scl syndrome because they occurred separated in time from each other, and in one patient even 6 years before the anti-PM/Scl syndrome, and one case as late as 1 year after the onset of the disease. Lazzaroni et al. reported the frequency of malignancies was not significantly higher in anti-PM/Scl positive SSc patients than in anti-PM/Scl negative controls from the EULAR Scleroderma Trials and Research group (EUSTAR) database (19). More cases are needed to confirm any associations between the malignancy and the disease.

Interstitial lung disease (ILD) is frequently associated with DM/PM and SSc, and ILD significantly contributes to morbidity and mortality, so it is important to clarify the clinical characteristics of ILD in such patients. As noted previously (6, 13, 14, 16, 17, 20), the frequency of ILD identified concurrently with anti-PM/Scl antibodies positive patients was from 50 to 86%. The predominant clinical feature of patients with PM/Scl antibodies was ILD (nearly 90%) in this study. Furthermore, the patterns of ILD basis of HRCT or lung biopsy were most commonly NSIP, followed by UIP and OP; LIP was rarely identified in this study. Similar to our cohorts, the most common radiologic pattern in other cohorts was NSIP (17, 20, 21).

More importantly, more than a quarter of patients with anti-PM/Scl antibodies were suffering from ILD alone. This result suggested that anti-PM-Scl positive patients can present with ILD without overt extrapulmonary manifestation. It is not clear whether signs of CTD may be developed later in some patients. We are concerned that “idiopathic” ILD might be diagnosed without examination of anti-PM/Scl antibodies because some patients with the antibodies show interstitial pneumonia without any symptoms suggestive of CTD. Although such patients have no apparent symptoms, interstitial pneumonia might be associated with autoimmune disease. It is, therefore, necessary to screen for such antibodies in patients with “idiopathic” ILD in the absence of signs of CTD.

To explore whether patients with anti-PM/Scl-75 or anti-PM/Scl-100 have distinct clinical features, this study compared the difference between two groups, the results demonstrated that more than a quarter of patients with anti-PM/Scl-75 had fever during the course of the disease, and the levels of inflammatory indicators such as ESR and CRP were much higher than that of the PM/Scl-100 group. This suggested that patients with anti-PM/Scl-75 antibodies were more prone to inflammatory reactions, but the cause of the fever could also have been associated with infection. Hanke demonstrated that anti-PM/Scl-75 antibodies were detected more frequently in younger and more active patients with joint contractures, and anti-PM/Scl-100 antibodies were associated with creatine kinase elevation; however, gastrointestinal involvements were observed less frequently (22). In our cohort, muscle weakness and dysphagia mainly occurred in the anti-PM/Scl-75 positive group, while Raynaud's phenomenon mainly occurred in patients with anti-PM/Scl-100, however, there is no statistically significant conclusion due to a small number of cases. Another study also showed that similar clinical features between anti-PM/Scl-75 and anti-PM/Scl-100 (6).

Similar to prior reports that Ro-52 antibodies have been detected commonly in antisynthetase syndrome (ASS) (23), 40% of patients in our cohort had Ro-52 antibodies. Furthermore, we analyze the difference between anti-PM/Scl associated syndrome with and without Ro-52, the result showed that patients carried with Ro-52 were prone to DM, and the frequency of muscle weakness and ILD in the Ro-52 group seemed to be higher than the group without anti-Ro52. De Lorenzo and colleagues also reported anti-PM/Scl patients who were positive for Ro-52 showed more severe muscle involvement (6).

Most of the patients with PM/Scl-ILD responded well after immunotherapy, especially for patients with NSIP, but some of them were able to survive with the ongoing disease even without immunotherapy. This seems to show that a chronic stable clinical course in patients with PM/Scl-ILD is a possible manifestation. In this study, antilung fibrosis drugs alone are not effective in NSIP patients with positive PM/Scl antibodies; the disease still belongs to the category of CTD, so immunotherapy may be needed to control the disease. Since UIP is no reversible inflammation, it is usually treated with antifibrotic drugs alone to slow down the progress of the disease. Some patients eventually need lung transplantation. However, a previous study showed that the prognosis of CTD-UIP was better than idiopathic pulmonary fibrosis (IPF)-UIP (24). In this study, of the 4 patients with UIP, 1 received antifibrotic drugs and 3 were given immunotherapy, but only one improved in the follow-up. Due to the small number of UIP cases included in the study and the short follow-up time, whether the treatment response and prognosis of PM/Scl antibody-related UIP were better than IPF-UIP, more cases are needed to observe the prognosis in a longer follow-up. Other studies showed a clear link between Th-17 cytokines and CTD-ILD (25, 26). This suggested that Th17 may be a potential target for the treatment of ILD. A higher prevalence of Covid-19 was also found in CTD patients with ILD (27). The Covid-19 pandemic may have a deleterious impact on patients with CTD. Therefore, we recommend that these patients be vaccinated against COVID-19. This study has several limitations. Only a relatively small number of patients were included, and although follow-up was complete, it was a relatively short time interval in some patients. Because this was a retrospective study, complete clinical data were not available for every patient. Finally, antibody detection using western blotting instead of immunoprecipitation may have resulted in false negatives or false positives, therefore missing some anti-PM/Scl antibodies positive patients.

In conclusion, this study demonstrated that patients with anti-PM/Scl had significant heterogeneity and involved skin, muscle, joint, and lung. Half of the patients had DM/PM, and anti-PM/Scl-75 antibodies are more dominant in anti-PM/Scl, but there was no significant difference compared with anti-PM/Scl-100. ILD was the main and most important clinical feature, NSIP was the main pattern of ILD. It could be helpful to examine anti-PM/Scl antibodies in interstitial pneumonia even if signs of myositis or CTD are absent. Most responded well to immunotherapy, but some patients were easy to relapse. Further studies are needed to clarify the details of anti-PM/Scl syndrome.

## Data Availability Statement

The raw data supporting the conclusions of this article will be made available by the authors, without undue reservation.

## Ethics Statement

The studies involving human participants were reviewed and approved by Research Review Committee (RRC) and the Ethical Review Committee (ERC) of the China-Japan Friendship Hospital (IRB number is 2016-117). Written informed consent for participation was not required for this study in accordance with the national legislation and the institutional requirements.

## Author Contributions

GW took responsibility for the integrity and the accuracy of the data analysis. All authors contributed to the article and approved the submitted version.

## Funding

This study was supported by the Academy Level Project of China-Japan Friendship Hospital (2019-1-QN-8).

## Conflict of Interest

The authors declare that the research was conducted in the absence of any commercial or financial relationships that could be construed as a potential conflict of interest.

## Publisher's Note

All claims expressed in this article are solely those of the authors and do not necessarily represent those of their affiliated organizations, or those of the publisher, the editors and the reviewers. Any product that may be evaluated in this article, or claim that may be made by its manufacturer, is not guaranteed or endorsed by the publisher.
